# Evaluation of the Impact of an Enzymatic Preparation Catalyzing the Decomposition of Raffinose from Poor-Quality Beets during the White Sugar Production Process

**DOI:** 10.3390/molecules29153526

**Published:** 2024-07-26

**Authors:** Andrzej Jaśkiewicz, Alina Kunicka-Styczyńska, Andrzej Baryga, Radosław Michał Gruska, Stanisław Brzeziński, Beata Świącik

**Affiliations:** Department of Sugar Industry and Food Safety Management, Faculty of Biotechnology and Food Sciences, Lodz University of Technology, Wólczańska 171/173, 90-530 Lodz, Poland; andrzej.jaskiewicz@p.lodz.pl (A.J.); andrzej.baryga@p.lodz.pl (A.B.); radoslaw.gruska@p.lodz.pl (R.M.G.); stanislaw.brzezinski@p.lodz.pl (S.B.); beata.swiacik@p.lodz.pl (B.Ś.)

**Keywords:** sugar beet, white sugar, raffinose, α-galactosidase, sugar raw juice

## Abstract

The study investigates the efficacy of an enzymatic preparation primarily with α-galactosidase activity for improving the quality of white sugar from poor-quality sugar beets. Focused on overcoming raffinose accumulation challenges in sugar beets, especially those harvested prematurely or stored for extended periods, an innovative exploration of enzymatic application in an industrial setting for the first time was conducted. By integrating theoretical calculations and experimental data, the findings reveal that α-galactosidase preparation notably diminishes raffinose content in beet juice, thus enhancing the sucrose yield and overall sugar quality. A reliable method to process lower-quality beets, promising enhanced efficiency in sugar production, was presented. The study also highlights the economic benefits of incorporating enzyme preparation into the production process, demonstrating a notable return on investment and underscoring the potential of enzymatic treatments to address industry challenges.

## 1. Introduction

The quality of sugar beet, a key raw material in white sugar production, is significantly influenced by its condition at the time of harvest. Sugar beets (*Beta vulgaris* L. subsp. *vulgaris*) are a seasonal and perishable plant material with a relatively short optimal technological harvesting period. This period’s brevity often necessitates the processing of both prematurely harvested and long-stored beets, leading to a range of quality issues. Moreover, storage conditions, where there are factors such as respiration, decay, and physical breakdown, contribute to a reduction in the sucrose yield [1,2,3].

A critical challenge in sugar beet storage is the accumulation of alpha-amino acid nitrogen, reducing substances (invert, primarily glucose, and fructose), and notably, raffinose [4,5,6]. The latter, an oligosaccharide, increases in concentration during storage and is particularly subjected to low temperatures [7]. Raffinose (C_18_H_32_O_16_) is a trisaccharide consisting of one molecule of galactose joined to a molecule of sucrose by an alpha 1–6 glycosidic bond [8]. Raffinose, initially found in small quantities at the time of sugar beet harvesting, tends to increase during the storage period. Typically, the concentration of raffinose at the point of harvest ranges from 0.1% to 0.8% of dry matter [9,10,11]. During the harvest period, the concentration of raffinose is considerably higher in the crown of the plant, with reported levels being twice as high as those found in the root below it [6,11].

The beet heads accumulate significant amounts of raffinose at temperatures below zero degrees Celsius [10,11]. Studies suggest that the elevated levels of raffinose in beet crowns could also be linked to plant respiratory activity. The reduction in temperature to near-freezing might trigger raffinose synthase production, leading to the transformation of respiratory by-products into raffinose [12]. Another investigation revealed that storing raw sugar beets under 4 °C leads to a rise in raffinose levels, indicating that sugar beets kept at 2 °C exhibit a 19% higher raffinose level compared to those stored at 6 °C [13]. The amount of raffinose in sugar beets is influenced by cultivation conditions, the timing of harvesting, and the duration of storage. Delayed harvesting and storage of the crop towards the end of the sugar campaign, especially if it exceeds 14 days, leads to a doubling of the raffinose content. Beets harvested in early September show a raffinose level of 4.2 g/kg in the crown and 3.4 g/kg in the root, whereas those harvested in late October demonstrate 8.9 g/kg in the crown and 4.1 g/kg in the root [13].

In sugar technology, raffinose interferes with the crystallization process by integrating into the sucrose crystal lattice or by increasing the viscosity of the syrup, thereby reducing the mobility of sucrose molecules and slowing down their crystallization rate. This effect is particularly pronounced at high concentrations of raffinose, leading to a reduction in the yield and purity of crystallized sucrose [14]. Furthermore, the presence of raffinose in sugar solutions contributes to the overall non-sucrose component, which complicates the sugar refining process and requires additional steps for removal or mitigation.

Raffinose contributes to the cloudiness of sugar solutions by increasing the solute concentration and, consequently, the refractive index. The effect of raffinose on solution cloudiness is also related to its interactions with other compounds in the sugar solution, such as proteins and minerals, which can further exacerbate the cloudiness [15,16]. The raffinose stability in an alkaline environment during liming processes causes it to go through all stages of sugar production and accumulate unchanged in molasses [17]. Therefore, the hydrolysis of raffinose seems to be a solution to reduce its level during the technological process. To date, only a few scientists have investigated the process of raffinose hydrolysis in molasses [16,18]. Despite the use of raffinose hydrolysis in the processing of pulses [19], in the production of protein hydrolysates [20] and enzymatic hydrolysis to melibiose and fructose, using invertase, so far, enzymatic hydrolysis has not been applied in sugar production process [21]. Plant-derived α-galactosidase for the hydrolysis of galactosyl-saccharides, including melibiose, raffinose, stachyose, and guar gum working in a broad spectrum of pH (US20060084163A1) [22] may also serve a potential factor for raffinose decomposition in sugar production. A technological solution for the use of α-galactosidase in sugar technology has been proposed to remove raffinose from molasses (US4036694A) [23]. To the best of our knowledge, the application of α-galactosidase, specifically at the extraction stage, has not been commonly adopted in the sugar industry. In addition to solving technological problems during the production process, alternative methods are also being sought to increase the efficiency of white sugar production and remove undesirable compounds, including raffinose [24]. One of the alternative methods of counteracting the effects of poor-quality beets is the use of enzymatic treatments, such as dextranase. However, enzymes like dextranase often have limitations, including limited shelf life, sensitivity to process conditions, and the need for additional equipment, which can increase production costs and complicate the sugar production process. While dextranase addresses issues related to dextran content, it does not affect raffinose levels, which are a significant concern in poor-quality beets [25,26,27]. In this study, we focus on α-galactosidase, which specifically targets raffinose, a key problematic compound in poor-quality beets. Unlike dextranase and other enzymes that may affect various sugar components, α-galactosidase offers a targeted approach to improving sugar quality and yield by directly addressing the raffinose issue. The use of α-galactosidase has the potential to overcome some of the limitations associated with other enzymatic treatments in sugar processing. Eliminating raffinose will also improve the crystallization process, preventing the formation of uneven, needle-like crystals [18], which will minimize the formation of distorted crystal forms during the first and the second boiling stages at this step of production. This improvement in crystal uniformity is crucial for achieving high-quality sugar products and optimizing the crystallization process. Moreover, it improves the efficiency of crystal separation during centrifugation. The enzymatic breakdown of raffinose leads to a more uniform crystal formation and reduces the viscosity of the sugar solution. As a result, the mechanical load on centrifuges may be diminished, reducing abrasion and potentially extending the operational life of the machinery. Additionally, a more efficient separation process can lead to higher sugar yields and reduced processing times, contributing to overall improved productivity [8,26,28]. The raffinose decomposition is facilitated by α-galactosidase, which acts by cleaving the galactosyl component from raffinose, resulting in the formation of galactose and sucrose [10,12]. α-Galactosidase (α-D-galactoside galactohydrolase, formerly known as melibiase, EC 3.2.1.22) hydrolyzes α-1,6-glycosidic bonds, leading to the release of terminal non-reducing α-D-galactose molecules from α-D-galactosides (small galactosidic oligosaccharides–melibiose, raffinose, and stachyose), galactopolysaccharides, and galactolipids. This enzyme also demonstrates activity on α-D-fructosides [16]. The problem of maintaining the effectiveness of the α-galactosidase under the production process is the time, temperature, and pH. Taking into account our previous research (data unpublished), we propose the use of the enzyme at the initial production stage in the process of sugar extraction from sugar beets. In a series of laboratory experiments testing various combinations of temperature (30, 55, and 75 °C) and incubation time (10, 20, and 30 min), we determined the conditions that maximized raffinose hydrolysis while minimizing the impact on other sugar components. The temperature 55 °C, pH 5.1, and 10 min incubation time were set as the most optimal for α-galactosidase activity in sugar beet juice. Enzymatic hydrolysis at the extraction stage is an innovative solution that has not been widely used on an industrial scale.

The study aims to apply the enzymatic commercial preparation with α-galactosidase activity at the onset of the technological process in sugar production, utilizing full-scale industrial sugar extraction and processing equipment at an operational sugar factory. This approach allows for the evaluation of the method under authentic industrial conditions, reflecting the realities of large-scale sugar production. The findings of this study were elaborated both at the laboratory level using in situ tests and on an industrial scale. Their applicability and innovation potential lie in enhancing the efficiency and quality of white sugar production from poor-quality beets. Moreover, the usefulness of enzymatic preparation against raffinose can contribute to the total utilization of deteriorated sugar beets and the reduction in waste plant biomass from sugar production.

## 2. Results and Discussion

### 2.1. Model Test with Enzyme Preparation

To evaluate the activity of an enzymatic preparation designed for the degradation of raffinose in raw sugar beet juice, laboratory model studies were conducted. The applied commercial enzymatic preparation was based on a crude extract of *Aspergillus niger* growth culture with α-galactosidase as the main compound. The utility of employing *A. niger* crude extracts in biotechnological applications of α-galactosidase stems from the mold ability in an extracellular production of this enzyme [29].

**Figure 1:**
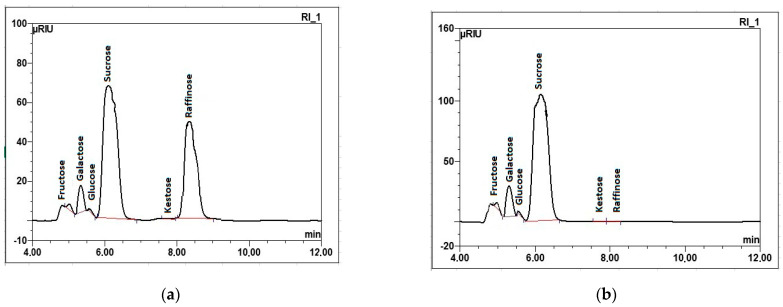
HPLC chromatograms of free carbohydrates in raw sugar beet juice from good-quality sugar beets with raffinose (**a**) and after incubation with enzyme preparation at 55 °C for 10 min (**b**), the red lines represent peak integration.

In situ tests of raw juice originating from a sugar factory made of good-quality sugar beet roots and fortified with raffinose were used and subjected to the enzyme preparation at 55 °C, pH-5.1, within 10 min, due to the previously set optimum operation of the enzyme preparation (unpublished data) and considering the parameters of the sugar production process as it was described in the Introduction. The results demonstrated the specificity and potency of the enzymatic preparation in raw juice (Table 1, Figure 1). The concentration of galactose and sucrose increased by 67.4% and 71.4%, respectively, due to enzymatic hydrolysis. Simultaneously, the raffinose level underwent a dramatic decrease of 99.5%, showcasing a significant breakdown by the enzyme. The experimental results closely matched the theoretical predictions, confirming the efficacy of the enzymatic treatment. However, slight discrepancies observed in the galactose levels suggest potential areas for further optimization [30].

The enzymatic cleavage of raffinose has been demonstrated using various enzyme preparations, including recent studies on the application of α-galactosidase in food processing. For example, Divakar investigated the use of α-galactosidase to reduce antinutrient content, including raffinose, in plant-based flours [31]. The study conducted by Lahuta et al. [30] on the germination of winter vetch seeds under the influence of an α-galactosidase inhibitor provides evidence of the enzyme’s role in natural physiological processes. The accumulation of raffinose in the presence of the inhibitor, alongside a corresponding decrease in galactose and sucrose levels, highlights the natural occurrence of this enzymatic breakdown in plant metabolism. The increased raffinose level in poor-quality sugar beets may be a result of a deficiency in plant cell metabolism. The use of α-galactosidase in breaking down oligosaccharides in jackfruit bulbs and seed flour, leading to a significant decrease in raffinose content, was also demonstrated [31]. Mabinya et al. [32] provide insight into the enzymatic breakdown of raffinose by α-galactosidase in *Erwinia chrysanthemi* into galactose and sucrose. In enzyme-aided treatment of fruit juice (watermelon), the increase in sucrose and galactose was found to result from the conversion of both raffinose and other sugars or their release from larger carbohydrate complexes [33]. Our results demonstrated the specificity and potency of the enzymatic preparation in raw juice (Table 1, Figure 1). The concentration of galactose increased from 2.64 ± 0.02% to 4.42 ± 0.05% (an increase of 67.4%), and sucrose increased from 8.49 ± 0.02% to 14.55 ± 0.03% (an increase of 71.4%) due to enzymatic hydrolysis. Simultaneously, the raffinose level decreased from 8.69 ± 0.01% to 0.04 ± 0.01% (a decrease of 99.5%), showcasing a significant breakdown by the enzyme. This enzymatic activity has significant implications not only in industrial applications, where the removal of raffinose is desired to improve the nutritional quality of food products but also in biological systems, where sugar metabolism is essential for various physiological processes. The enzymatic breakdown of raffinose by α-galactosidase in plant or plant-derived products, resulting in increased levels of galactose and sucrose, is in agreement with our studies.

The stability of glucose, fructose, and kestose levels in raw sugar beet juice after the enzymatic treatment suggests specific preparation activity that selectively targets other sugars while leaving kestose unchanged. Recent studies have shown that α-galactosidases exhibited high specificity for their substrates. For instance, Zhang et al. [16] found that α-galactosidases primarily hydrolyze α-1,6-linked galactose residues in oligosaccharides and polysaccharides but do not act on β-linked galactosides or α-glucosides. Kestose, being a fructooligosaccharide, is unlikely to be a direct target of α-galactosidase due to its structural composition. Yeo and Liong [34] discuss the increase in α-galactosidase activity in probiotics when supplemented with prebiotics in soymilk, which leads to enhanced hydrolysis and the utilization of soy oligosaccharides raffinose and stachyose, leaving kestose intact.

### 2.2. Technological Tests

Technological tests were conducted in the sugar factory, where enzyme preparation was applied during the extraction of raw beet root juice at the end of the sugar campaign in December 2022 and January 2023, according to the experimental design described in point 3.5. The quality of beets significantly impacted the chemical composition of both the raw juice and the final product, which was the resultant sugar. The data presented in Table 2 provide the parameters of good- and poor-quality beets as well as raw juice and sugar that originated from them.

#### 2.2.1. Chemical Quality of Sugar Beet

From the perspective of white sugar production technology, the efficiency of processing sugar beets and the quality of the final product are significantly influenced by parameters in the beet root: the sucrose, ash, and insoluble matter content. The results are further categorized based on the quality of the beets, with distinctions between good-quality and poor-quality beets.

**Figure 2:**
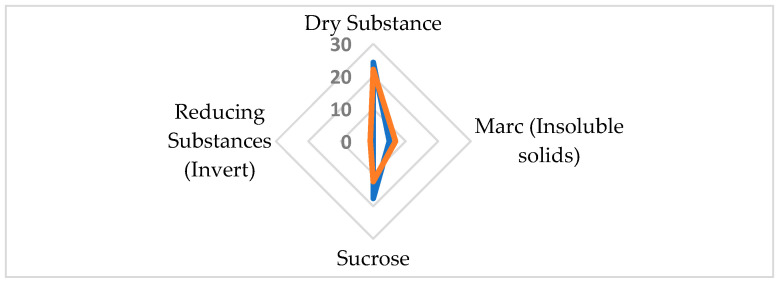
Radar chart comparing main chemical parameters (%) suboptimal for the technological process of white sugar production, orange—poor-quality sugar beets; blue—good-quality sugar beets.

According to the parameters presented in Table 2, good-quality sugar beet raw material met the requirements in terms of the technological value of beets sent for processing in the sugar factory [35,36,37,38]. The chemical quality of the examined sugar beet roots marked as poor quality differs substantially from that typical for beets of appropriate quality in four out of nine studied parameters: dry substance, marc (insoluble solids), sucrose, and reducing substances (invert sugar) content (Figure 2).

The dry substance content of good-quality beets was found to be 24.31%, a level typical for healthy beets (24–25%) [1,2,3,4] and higher by 2.2% than the poor-quality ones. Good-quality beets were characterized by a lower water content and a higher concentration of valuable components [16,17,18]. The change in the dry substance may be an effect of more intense water loss due to drying and the consumption of sucrose in respiration processes during beet storage [8].

The water-insoluble components of sugar beet (marc) of good-quality beets were 4.97%, which falls within the literature values for healthy beets (4.0–5.0%) [8]. In contrast, bad-quality beets expressed a higher marc content (6.82%). This is contrary to some literature showing the marc content decreased by 10% [8]. In turn, in another study, an increase in marc was confirmed both in beets of deteriorated quality and in stored beets [6]. However, there are reports that degraded beets have a higher marc content compared to healthy beets, which makes sucrose extraction more difficult [8,39]. The amount of marc in different beet varieties is usually linked to their sucrose content, as varieties with higher sucrose content tend to have smaller yet more abundant parenchymal cells, leading to a greater quantity of cell wall compounds [6]. The amount of marc in the root was closely and negatively related to its vulnerability to damage and, consequently, to pathogen infestation, which may correlate with sugar losses during storage [6].

Our results show that the sucrose content varied significantly based on the quality of the beets. The sucrose level in good-quality beets was estimated as 17.81%, while in poor-quality ones, it was 5.5% lower. During storage, part of the sucrose that accumulates in the beet cells is metabolized to maintain their survival [40,41]. After removing the root tops, the synthesis of sucrose in sugar beet roots stops, but the chemical and biological processes continue. During respiration, heat is generated, and energy is released at the expense of the sucrose oxidation [42]. Cell invertase activity results in sucrose degradation, increasing glucose and fructose levels and leading to the crystallization worsening and a decrease in the molasses content. By increasing the temperature by 10 degrees, sucrose losses doubled. Anaerobic storage of sugar beets can halve the formation of carbon dioxide, but intermolecular respiration increases, and sucrose is used to form non-sucrose compounds [42]. Surface damage of sugar beet roots results in an intensification of sucrose loss due to an increased invasion of mold [5]. Mechanical injuries of sugar beet lead to wound healing processes, which require sucrose decomposition [43].

Sugar beet roots are stored for several weeks post-harvest before being processed in sugar factories. During this storage period, there can be a loss of sucrose and the accumulation of inverted sugar, which decreases the final sugar yield and processing quality [10]. Gippert et al. [10] investigated the primary and secondary metabolites of six sugar beet varieties during storage. They found that beet varieties performing well during storage expressed higher pools of certain free amino acids (glutamine, proline, alanine, arginine, and aspartate) at harvest [10]. The initial composition of the beets at the time of harvest may affect their storability and subsequent quality after storage.

Good-quality beets in our studies were characterized by a statistically significant fewer reducing substances (invert) (0.08%) compared to poor-quality beets (0.92%). This suggests a higher purity of sugar in good-quality beets, which is consistent with the literature data of inverted levels in healthy beets (0.02–0.10%) [8]. The long-term storage and the infestation with root rots result in a marked increase in the inverted sugar concentration with detrimental consequences for processing [35].

The quality of sugar beets can also be influenced by the time of harvest. Beets left in the ground beyond the optimal harvest period undergo biochemical changes, leading to a reduction in sucrose content, dry substance, and potassium levels [37]. Cold stress leads to an increase in raffinose content in sugar beet taproots, suggesting its protective role against freezing damage [11].

#### 2.2.2. Chemical Quality of Raw Juices

In the context of white sugar production, the quality of raw juice plays a pivotal role in the efficiency and quality of the final product. This aspect is frequently emphasized in technological and scientific literature due to its direct correlation with the efficiency of processing operations and the quality of the sugar [44,45].

Firstly, the chemical composition of raw juice after extraction of beet cossette, including the concentration of sucrose, minerals, and other organic and inorganic compounds, significantly influences the sugar crystallization process. High-quality juice contributes to more effective crystallization, which, in turn, impacts the sugar production yield [39].

**Figure 3:**
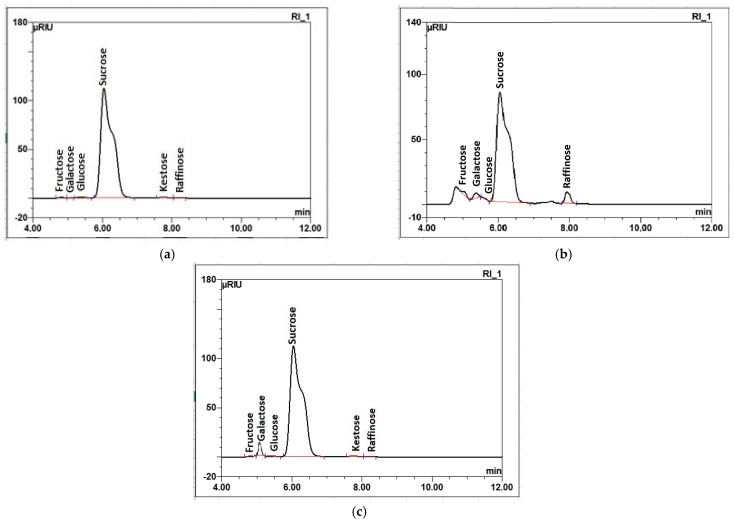
HPLC chromatograms of free carbohydrates in raw sugar beet juice from (**a**) good-quality sugar beets, (**b**) poor-quality sugar beet, (**c**) poor-quality sugar beets after enzymatic treatment, the red lines represent peak integration.

The qualitative composition of individual carbohydrates in raw juices produced in our experiments was in line with the literature data. Sucrose is the dominant sugar in the free carbohydrate profiles of all the tested raw juices (Table 2, Figure 3), which is in agreement with the literature data [9].

**Figure 4:**
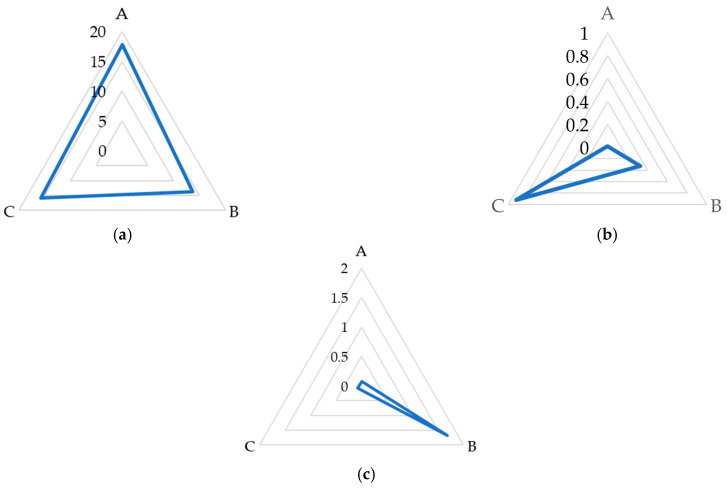
Radar chart comparison of sucrose (**a**), galactose (**b**) and raffinose (**c**) (%) from raw juices from A—good-quality sugar beets; B—poor-quality sugar beet; C—poor-quality sugar beets with enzymatic preparation treatment.

The quantitative carbohydrate composition in raw sugar beet juices exhibited significant variability in the inherent beet quality and the enzymatic treatments applied during extraction. High-quality beet juice, characterized by a sucrose concentration of 17.81% and low raffinose level (0.08%), exemplified optimal conditions for sugar production. Conversely, juice derived from beets of inferior quality manifested a statistically significant diminished sucrose level of 13.61% alongside an augmented raffinose concentration increase of up to 1.68%. The elevation in raffinose suggests a suboptimal enzymatic conversion or degradation, which is potentially attributable to the inferior beet quality (Figure 3 and Figure 4).

**Figure 5:**
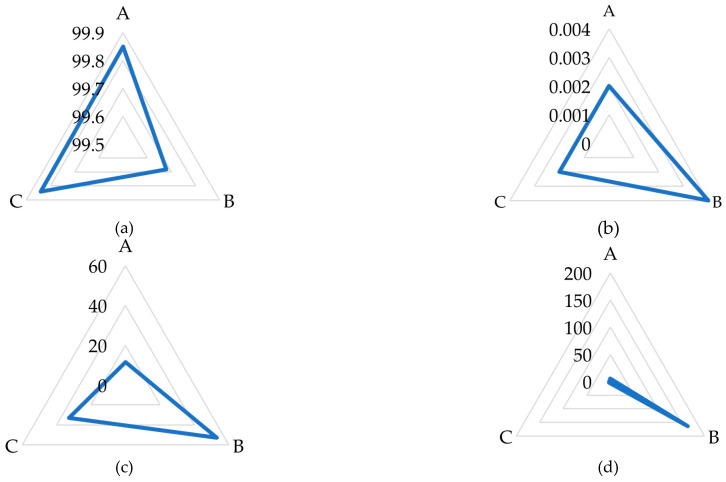
Radar chart comparison of various quality parameters of white sugar: (**a**) polarization (°Z), (**b**) concentration of reducing sugars (%), (**c**) color of sugar solution (IU_420_), and (**d**) insoluble matter (mg/kg) obtained from raw beet juices from A—good-quality sugar beets; B—poor-quality sugar beets; C—poor-quality sugar beets treated with enzymatic preparation.

The application of the enzyme preparation resulted in significant changes in the sugar composition of the raw juice (Figure 5b). The content of sucrose increased from 13.61 ± 0.34% to 14.79 ± 0.25% (an increase of 8.7%). Simultaneously, there was a substantial decrease in the raffinose from 1.68 ± 0.30% to 0.08 ± 0.02% (a decrease of 95.2%). The galactose level increased from 0.33 ± 0.04% to 0.92 ± 0.05% (an increase of 178.8%). These changes in absolute concentrations provide insight into the sugar transformations occurring during the enzymatic treatment. The decrease in raffinose (1.6%) closely corresponds to the combined increase in sucrose (1.2%) and galactose (0.6%). This suggests that the increase in galactose is primarily a result of raffinose decomposition rather than the breakdown of other sugars. Additionally, the detection of kestose (0.06 ± 0.02%) in enzyme-treated juice underscored the preparation specificity in oligosaccharide breakdown.

The enzymatic breakdown of complex carbohydrates in sugar beet pulp and its influence on the resulting raw juice composition is a topic of significant biotechnological interest. While there are limited publications on the use of α-galactosidase for raffinose degradation during sugar beet processing, some notable research has been conducted. Recent studies have demonstrated the potential of a thermostable, recombinant α-galactosidase for raffinose elimination from sugar beet syrup. Their study showed that the enzyme of *Bacillus stearothermophilus* was highly stable under process conditions, with a pH optimum close to neutrality, and its activity was not inhibited by D-galactose or sucrose [46]. Our study extends this concept by applying a soluble enzyme preparation directly in the extraction process of raw juice rather than treating syrup or molasses, which resulted in a high raffinose reduction (99%) on an industrial scale. Another research study has been limited to the use of α-galactosidase for the hydrolysis of raffinose in sugar beet molasses, which occurs at the later stage in the sugar production process [47]. Our approach, focusing on raw juice treatment, offers potential advantages in terms of process efficiency and sugar yield. The integration of enzymatic treatment into existing sugar production processes could significantly enhance efficiency and reduce waste, offering substantial economic and environmental benefits [26,27].

#### 2.2.3. Chemical Quality of White Sugar

The quality and purity of sugar derived from sugar beets are paramount in the sugar industry. The provided data delineate the distinct differences in sugar quality parameters between good-quality beets, poor-quality beets, and those obtained with the enzyme preparation (Table 2, Figure 5).

The implementation of the enzyme preparation to poor-quality beet juice significantly ameliorates chemical sugar parameters, nearly aligning polarization (99.84 °Z) with that of good-quality sugar. There was an increase in polarization after the enzyme preparation of 0.17% compared to sugar obtained from poor-quality beets. Although moisture content increased by 12.5% in sugar after the application of an enzyme preparation to the sugar from poor-quality beet without enzyme, it maintained low levels of reducing sugars (0.002%).

Notably, while ash content rose over three times relative to the sugar from good-quality beets, it was 0.65% lower than that from untreated poor-quality beets, indicating reduced mineral contamination. Color parameters also improved, although not to the level of good-quality beet sugar, highlighting the enzyme’s efficacy in enhancing sugar quality from lower-grade beets (Figure 5).

Good-quality beet sugar was characterized by its superior polarization (99.85 °Z), indicating a high level of purity. A very low level of moisture (0.007%) and reducing sugars (0.002%) contribute to the sugar’s stability and prevent degradation. The low ash content (0.003%) and optimal color parameters reflect minimal mineral contamination and high sugar purity. A study conducted in the Toshka region of Aswan (Egypt) highlights the variability in sugar beet quality parameters, emphasizing the importance of beet in determining the final sugar quality [48]. Another study on honey quality, although not directly related to sugar beets, emphasized the importance of parameters like moisture, reducing sugars, and ash content in determining product quality [49].

These results elucidate the profound impact of elevated raffinose content on sugar quality, particularly on polarization, reducing sugars, and color parameters, underscoring the effectiveness of enzymatic treatment in improving sugar quality derived from poor-quality beets. This investigation not only demonstrates the critical role of enzymatic treatment in mitigating the adverse effects of raffinose on sugar quality but also contributes to the broader understanding of sugar beet processing and optimization for enhanced sugar production. While good-quality sugar beets naturally yield superior sugar, treatments by the applied enzyme preparation offer a promising avenue to enhance the quality of sugar derived from suboptimal-quality beets.

### 2.3. Confirmation of Experimental Data

Based on the sucrose and raffinose content determined in the raw sugar beet juice, the predicted amount of sucrose and galactose was calculated, assuming that the raffinose present decomposes due to enzymatic hydrolysis into sucrose and galactose as a whole. The influence of the enzyme preparation on the carbohydrate composition of sugar beet juice was further corroborated by theoretical calculations. It was assumed that the hydrolysis of raffinose produces equimolar amounts of sucrose and galactose, and these were subsequently converted into masses and percentage concentrations for comparison with the initial data [50].

**Figure 6:**
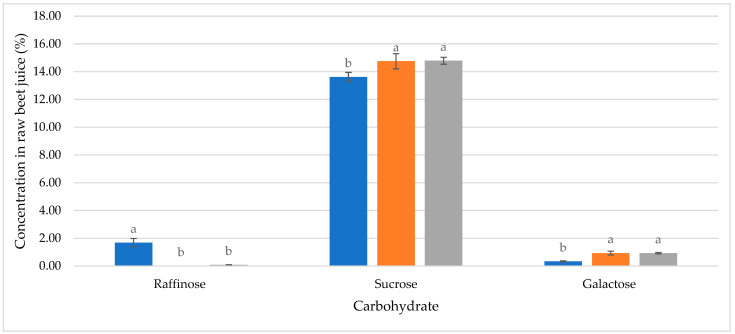
Comparison of experimental results with theoretical enzymatic raffinose hydrolysis efficiency in raw sugar beet juice; blue—raw juice without enzymatic treatment; orange—theoretical values after enzymatic treatment; grey—experimental data after enzymatic treatment; a and b—the same letters indicate no statistically important differences between the same carbohydrate in different experiments (*p* < 0.05).

As presented in Figure 6, the predicted values provide a range for the expected sucrose and galactose percentages in the juice.

The mean value of sucrose content in raw juice treated by enzyme preparation was equal to 14.79 ± 0.25% (Table 2), aligned with the theoretically forecasted range of 14.70 ± 0.55%. Similarly, the experimental mean value for galactose content was 0.92 ± 0.05%, which fits precisely within the theoretical forecasted range of 0.90 ± 0.14% (Figure 6).

Such a methodological approach allowed us to validate the experimental findings and provide a robust framework for predicting the influence of enzymatic treatments on the carbohydrate composition of sugar beet juice. Comparative studies with other enzymatic treatments or alternative methods could further validate the efficacy and cost-effectiveness of the α-galactosidase preparation [51].

### 2.4. Effectiveness of the Use of Enzyme Preparation in the Industrial Scale Application

To confirm the cost-effectiveness on the industrial scale of the enzyme preparation application, a calculation was provided. Based on sugar producers’ reports [52] the following assumptions were set up:

The sugar factory in Poland processed an average of 7716 tons of sugar beets per day during the 2022/2023 campaign [52]. The campaign lasted 108 days, with an average sugar yield of 14.3% [52]. Sugar yield, an indicator of the efficiency of the technological process in a sugar factory, is defined as the amount of sugar obtained from a specific quantity of sugar beets during the production process and may vary from season to season. The ratio of beet mass to juice mass (“draft ratio”) was 1.0:1.1, so for 1 kg of beets, 1.1 kg of juice was generated [53]. The average price of sugar in January 2023 was 775 EUR/ton [54]. An enzymatic preparation was estimated to be applied during the last 40 days of the campaign, targeting difficulties in processing beets towards the end of November [52]. The average raffinose content in raw juice was 1.68%, and hydrolysis of 1 kg of raffinose released approximately 0.6786 kg of sucrose. The cost of 1 kg enzyme preparation, according to the producer, was 81 EUR.

○ Average sugar production: 7716 tons/day × 14.3% = 1103.38 tons/day.
○ Sugar uplift from raffinose hydrolysis: 1.68% × 0.68 = 1.14%.
○ Daily sugar gain from hydrolysis, including “draft ratio”: 1103.38 tons/day × 1.1 (draft ratio) × 1.14% = 13.84 tons/day.
○ Total sugar gain during enzymatic application period: 13.84 tons/day × 40 days = 553.60 tons.
○ Value of additional sugar produced: 553.6 tons × 775 EUR/ton = 429,040 EUR.
○ Cost of the enzyme preparation (according to data received from the producer):
  ▪ 1.5 kg × 24 h × 40 days = 1440 kg.
  ▪ 1440 kg × 81 EUR = 116,640 EUR.
○ Net profit: 429,040 − 116,640 = 312,400 EUR.

The application of enzyme preparation on an industrial-scale sugar beet processing has demonstrated significant economic benefits. The additional sugar yield of 553.6 tons during the last 40 days of the campaign resulted in a net profit of 312,400 EUR after accounting for the enzyme costs. This highlights the cost-effectiveness of using enzymatic treatments to enhance sugar recovery efficiency and maximize revenue in sugar beet processing.

## 3. Materials and Methods

### 3.1. Sugar Beets, Raw Juices, and White Sugar

The studied materials were sugar beets (*Beta vulgaris* L. subsp. *vulgaris*), a classic variety that originated from a plantation of a Polish sugar factory located in the south of the country. Good-quality beets were harvested at the beginning of the campaign period (October 2022) and were fully ripe for processing. Poor-quality beets were processed at the end of the beet root campaign (December 2022 and January 2023), and previously stored mounds were subjected to weather-changing factors. For one sample, 50 kg of roots were taken at random, milled, and blended to ensure its homogeneity. A total of 5 samples were collected and analyzed for each condition to ensure statistical reliability.

Raw juice and white sugar were obtained from a Polish sugar factory during the 2022/2023 campaign after the extraction process. Efforts were made to sample beets, juices, and sugars to maximize certainty that the juice and white sugar originated from the specific beets undergoing processing. The amount of raw juice taken for analysis was 4 L per one analysis, and the amount of white sugar was 5 kg per one analysis. All analyses were performed in 5 replications.

### 3.2. Chemicals and Reagents

The following standards and reagents were used: sucrose (≥99.5 HPLC purity, Fluka Honeywell International Inc., NJ, USA), raffinose pentahydrate (≥97.0 HPLC purity, Merck KGaA, Darmstadt, Germany), fructose (D-(-) fructose (≥99.0 HPLC purity, Fluka Honeywell International Inc., NJ, USA), anhydrous glucose p.a. (≥99.5% sum of enantiomers HPLC purity, Sigma-Aldrich St. Louis, MO, USA), arabinose (HPLC purity, Fluka Honeywell International Inc., NJ, USA), galactose (Merck KGaA, Darmstadt, Germany), acetonitrile (HPLC purity, POCH, Gliwice, Poland).

### 3.3. Enzyme

Enzymatic commercial preparation Barygaza (Higenix, Łódź, Poland) was used for the hydrolysis of raffinose. The main ingredient of preparation is α-galactosidase. Enzyme activity declared by the producer was 3000 U/g, Patent no: PL227625. According to the manufacturer’s specifications, the enzyme has an optimal pH range of 5.0–7.0 and functions best at temperatures between 30 and 60 °C. The enzymatic activity, as reported by the manufacturer, was determined using raffinose as a substrate under the following conditions: temperature 50 °C, pH 5.5, with a reaction time of 10–20 min.

### 3.4. Laboratory Model Test on Raw Sugar Beet Juice

12 g of raffinose pentahydrate was added to 100 mL sugar beet raw juice of good quality (raw juice from the 3rd week of the campaign). A total of 1.5 mL (150 mg) of the enzyme preparation was added to 10 mL of juice and heated at 55 °C for 10 min. Analyses were performed in 5 repetitions. The raw juice was analyzed for raffinose, sucrose, glucose/galactose, and fructose using the HPLC method, according to the methodology described in Section 3.7.

### 3.5. Experiment Design in a Sugar Factory

**Figure 7:**
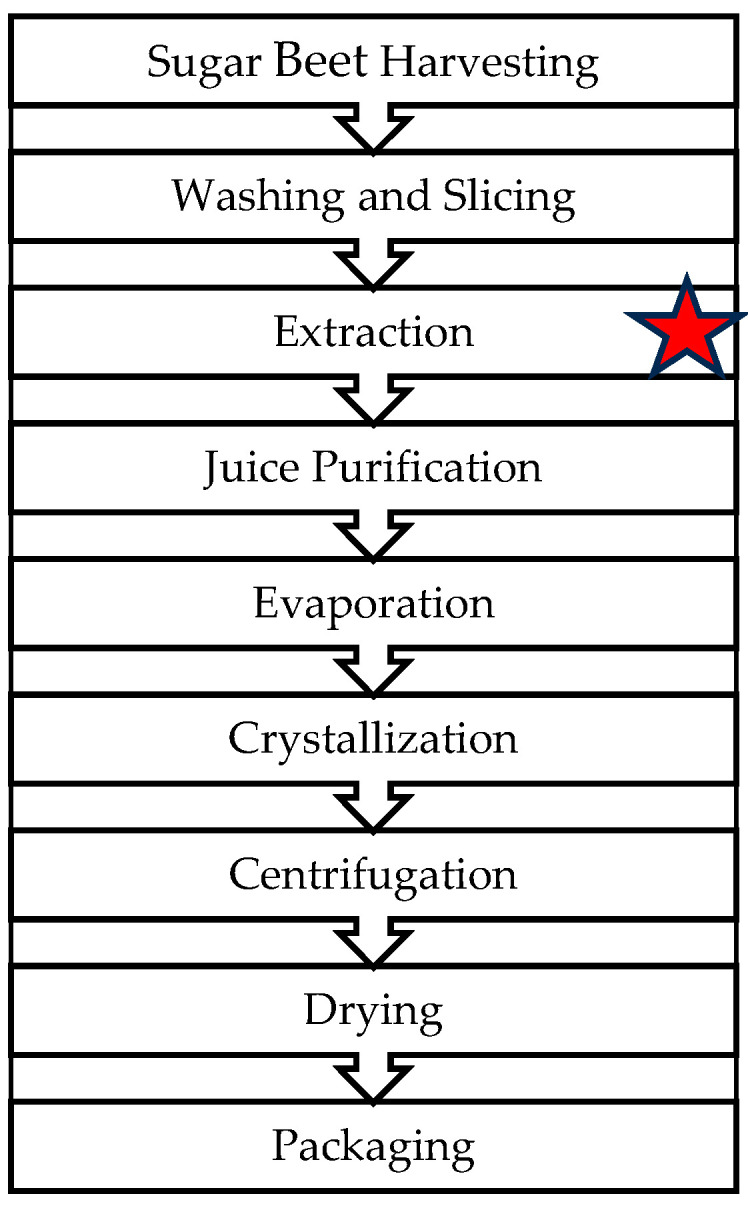
Simplified flow diagram of sugar production from sugar beets. The red star indicates the stage of extraction where the enzyme preparation was added.

The technological part of the experiment was performed in a Polish sugar factory located in the southern part of Poland. To check the possibility of technological improvement of the white sugar production process from poor-quality beet roots, enzyme preparation was applied during the stage of extraction. The extraction process was carried out in a tower extractor with the following parameters set: extraction 1:1 (1 ton of water was used per 1 ton of beets), amount of juice obtained 83.3–166.6 m^3^/h, temperature 55 °C, pH-5.1. The pH of 5.1 was maintained by the natural buffering capacity of the sugar beet juice components without the addition of an external buffering system. This pH level is typical for the extraction process in sugar production and is suitable for the optimal activity of the enzyme preparation used in this study. The enzyme preparation was added during the extraction of raw beet root juice. The dosage was calculated to be 75–150 mg of enzyme preparation per ton of beets processed. This corresponds to 2–4 L of enzyme preparation per 2000–4000 tons of beets per day, given the enzyme preparation’s density of approximately 1.2 g/mL. All analyses were performed in 5 replications. Figure 7 shows the sugar production process and the point of enzyme application.

### 3.6. Chemical Analysis of Sugar Beet Roots

Analysis of chemical quality parameters of sugar beet roots was performed according to Polish national and international standards (Table 3).

The analysis was conducted in five repetitions, and the results were presented as the average value with standard deviations.

### 3.7. Determination of Free Carbohydrates in Raw Sugar Beet Juice

The concentration of soluble carbohydrates was performed using high-performance liquid chromatography [9,59,60]. First, 2 g ± 0.1 g representative samples of juices were mixed with 10 mL of HPLC-grade redistilled water and incubated in a water bath at the temperature of 85 °C for 1 h with shaking. Afterward, the samples were cooled and centrifuged at 20 °C for 15 min at 4600 rpm. Then, 2 mL of supernatant was collected and filtered into HPLC measuring vials by nylon syringe filters (Chromacol, Herts, UK) with a pore size of 0.2 µm. HPLC-RI was used for analysis: UHPLC+ Dionex UltiMate 3000 system (Thermo Fisher Scientific Inc., Waltham, MA, USA) equipped with a refractive index detector (Shimadzu, Kioto, Japan). The following conditions were applied: Rezex column RPM Monosaccharide Pb2+ New Column, 8 µm, 7.8 × 300 mm; Eluent: 100% HPLC purity water; isocratic gradient; column preparation: 100% eluent in 20 min; analysis: 100% eluent in 35 min; flow rate: 0.6 mL/min; column temperature: 80 °C; injection volume: 10 µL; detection: Shodex R1–101, temperature 40 °C.

The chromatograms were analyzed using the HPLC system’s software. Peak integration was performed automatically, with the baseline set using a valley-to-valley integration method. This method defines the start and end of each peak at the lowest point between adjacent peaks. The red lines visible on the chromatograms (Figure 1 and Figure 3) represent these integrations. It should be noted that the chromatograms presented in Figure 1 and Figure 3 are primarily for illustrative purposes to show qualitative changes in sugar profiles, while quantitative results are provided in Table 1 and Table 2.

### 3.8. Chemical Analysis of Sugar

Table 4 presents the methodology of chemical evaluations of white sugar parameters.

The analysis was conducted in five repetitions. Results were presented as the average value with standard deviation.

### 3.9. Statistical Analysis

All analyses were conducted in five repetitions. Statistical evaluation was performed using Statistica 13.1 software (StatSoft, Cracow, Poland). In order to evaluate the normal distribution of groups, the Shapiro–Wilk test was performed. Additionally, Levine’s test was performed to confirm the homogeneity of variance, followed by a one-way analysis of variance (ANOVA) to compare the results and Tukey’s test to reveal pairs of groups that differed from statistical significance in terms of means. Significance was defined at *p* ≤ 0.05.

## 4. Conclusions

The research presented the innovative approach of enzyme preparation with α-galactosidase activity application in the sugar industry, highlighting its significant impact on enhancing raw juice and the resultant white sugar quality by an effective decomposition of raffinose into sugar beet juice. Within this process, not only was the raffinose content in raw juice decreased, but the sucrose yield was also increased, thus improving the overall quality of white sugar produced from poor-quality beets. The application of the enzyme preparation resulted in a noticeable increase in the sucrose and galactose content in the juice facilitated for sugar production processes.

α-Galactosidase involvement in the extraction step led to significant improvements in the chemical quality parameters of the produced white sugar, including polarization, reduced sugar percentage, the color of sugar solution, and insoluble matter content, testifying that enzymatic treatment can effectively mitigate the adverse effects of poor beet quality in sugar production.

Moreover, the use of the enzymatic preparation was profitable, providing a substantial net profit and demonstrating the cost-effectiveness of integrating α-galactosidase preparation into sugar production, especially when poor-quality sugar beet roots are processed. The analyses showed a significant return on investment, underscoring the financial viability of this technological advancement.

This research presents a promising approach for optimizing the efficiency and quality of sugar production, especially when dealing with lower-quality raw materials. The findings offer new perspectives for addressing challenges in the sugar industry and highlight the potential of enzymatic treatments in enhancing production outcomes. Due to the deteriorating quality of the raw material during the sugar campaign, our further work will focus on optimizing the dose of the enzyme depending on the degree of degradation of the sugar beet sent for processing.

## Figures and Tables

**Table 1:** Main carbohydrates in raw sugar beet juice fortified with raffinose and treated by enzymatic preparation (55 °C for 10 min.).

	Raw Sugar Beet Juice	
Carbohydrates, %	With Raffinose ^1^	With Raffinose after Enzymatic Preparation Treatment ^2^
Fructose	0.63 ± 0.01 ^a^	0.59 ± 0.03 ^a^
Glucose	0.17 ± 0.01 ^a^	0.15 ± 0.02 ^a^
Galactose	2.64 ± 0.02 ^b^	4.42 ± 0.05 ^a^
Sucrose	8.49 ± 0.02 ^b^	14.55 ± 0.03 ^a^
Trehalose	ND	ND
Kestose	0.02 ± 0.01 ^a^	0.02 ± 0.01 ^a^
Raffinose	8.69 ± 0.01 ^a^	0.04 ± 0.01 ^b^

^1^ raw juice from good-quality beet with 9% raffinose addition; ^2^ raw juice with 9% raffinose after incubation with enzyme preparation; ND, not detected; The same superscript letter in one raw indicates no statistically significant differences between carbohydrates (*p* < 0.05)

**Table 2:** Chemical quality parameters of sugar beet roots, content of free carbohydrates in raw sugar beet juices and quality of white sugar from various quality of beets.

	**Sugar Beet Roots**		**Raw Sugar Beet Juice**			**White Sugar**	
	**Parameter**	**%**	**Carbohydrates**		**%**	**Parameter**	
**Good quality**	Dry Substance	24.31 ± 0.04 ^a^	Fructose		0.03 ± 0.02 ^b^	Polarization (°Z)	99.85 ± 0.00 ^a^
	Marc (Insoluble solids)	4.97 ± 0.12 ^b^	Glucose		0.06 ± 0.01 ^a^	Moisture (%)	0.007 ± 0.000 ^c^
	Sucrose	17.56 ± 0.06 ^a^	Galactose		0.01 ± 0.01 ^c^	Reducing Sugars (%)	0.002 ± 0.000 ^b^
	Reducing Substances (Invert)	0.08 ± 0.01 ^b^	Sucrose		17.81 ± 0.30 ^a^	Ash (%)	0.003 ± 0.000 ^c^
	Soluble Ash Content	0.360 ± 0.003 ^b^	Trehalose		ND	Color in comparison with dry standard (IU_420_)	0.2 ± 0.0 ^c^
	Potassium	0.167 ± 0.002 ^a^	Kestose		0.02 ± 0.01 ^b^	Color of sugar solution (IU_420_)	11.50 ± 0.14 ^c^
	Sodium	0.0056 ± 0.0004 ^b^	Raffinose		0.08 ± 0.01 ^b^	Sulfite (mg/kg)	0.18 ± 0.00 ^c^
	Alpha-amino acid nitrogen	0.010 ± 0.000 ^a^				Insoluble Matter (mg/kg)	5.7 ± 0.4 ^b^
	Amide nitrogen	0.013 ± 0.002 ^a^				Turbidity (IU_420_)	5.4 ± 0.1 ^c^
						pH	6.01 ± 0.02 ^c^
**Poor quality**			**Without enzyme preparation**	Fructose	0.10 ± 0.02 ^a^	Polarization (°Z)	99.68 ± 0.00 ^c^
				Glucose	0.02 ± 0.01 ^b^	Moisture (%)	0.008 ± 0.000 ^b^
				Galactose	0.33 ± 0.04 ^b^	Reducing Sugars (%)	0.004 ± 0.000 ^a^
				Sucrose	13.61 ± 0.34 ^c^	Ash (%)	0.031 ± 0.001 ^a^
	Dry Substance	22.15 ± 0.03 ^b^		Trehalose	ND	Color in comparison with dry standard (IU_420_)	3.0 ± 0.1 ^a^
	Marc (Insoluble solids)	6.82 ± 0.16 ^a^		Kestose	ND	Color of sugar solution (IU_420_)	53.00 ± 0.24 ^a^
	Sucrose	12.35 ± 0.02 ^b^		Raffinose	1.68 ± 0.30 ^a^	Sulfite (mg/kg)	5.55 ± 0.09 ^a^
	Reducing Substances (Invert)	0.92 ± 0.02 ^a^				Insoluble Matter (mg/kg)	164.0 ± 4.1 ^a^
	Soluble Ash Content	0.450 ± 0.013 ^a^				Turbidity (IU_420_)	469.0 ± 0.9 ^a^
						pH	8.20 ± 0.08 ^a^
	Potassium	0.161 ± 0.009 ^b^	**With enzyme preparation**	Fructose	0.11 ± 0.01 ^a^	Polarization (°Z)	99.84 ± 0.00 ^b^
	Sodium	0.0068 ± 0.0003 ^a^		Glucose	0.01 ± 0.01 ^b^	Moisture (%)	0.019 ± 0.002 ^a^
	Alpha-amino acid nitrogen	0.009 ± 0.000 ^b^		Galactose	0.92 ± 0.05 ^a^	Reducing Sugars (%)	0.002 ± 0.000 ^b^
	Amide nitrogen	0.006 ± 0.000 ^b^		Sucrose	14.79 ± 0.25 ^b^	Ash (%)	0.011 ± 0.000 ^b^
				Trehalose	ND	Color in comparison with dry standard (IU_420_)	1.8 ± 0.1 ^b^
				Kestose	0.06 ± 0.02 ^a^	Color of sugar solution (IU_420_)	32.90 ± 0.21 ^b^
				Raffinose	0.08 ± 0.02 ^b^	Sulfite (mg/kg)	0.31 ± 0.01 ^b^
						Insoluble Matter (mg/kg)	3.1 ± 0.2 ^c^
						Turbidity (IU_420_)	6.0 ± 0.2 ^b^
						pH	6.69 ± 0.04 ^b^

IU, (ICUMSA Units) a unit of measurement for sugar color, determined based on the absorption of light at a wavelength of 420 nm; Z, polarization unit; ND, not detected; ^a, b, c^—the same superscript letter in one column indicates no statistically significant differences between the same parameters in the column (*p* < 0.05)

**Table 3:** Chemical analysis of sugar beet roots.

Parameter	Description	Methodology	Typical Range/Value, (%) [37]
Dry substance content	The amount of dry mass in sugar beet roots	ICUMSA GS2/3-1 (2011) [37,55]	24–25
Marc content	The amount of insoluble plant components remaining after juice extraction	Standard Method [37]	4.0–6.0
Sucrose content	The amount of sucrose	ICUMSA GS6-3 (1994) [56]	14.–19
Reducing sugars content (invert)	The level of reducing substances such as glucose and fructose, which are formed by the hydrolysis of sucrose	Berlin Institute Method [37]	0.02–0.1
Soluble ash content	The mineral content in beet juice	Conductometrically [37]	0.5–0.6
Amide nitrogen	Amide nitrogen content	National standard PN-EN 13342:2002 [57]	≤0.015
α-Amino acid nitrogen	The nitrogen content from amino acids	ICUMSA GS6-5 (2007) [58]	≤0.03
Metal concentration: sodium, potassium	Sodium and potassium content	ASA spectrophotometry, National Standard (FAAS) [38]	Na: 0.1–0.3, K: 0.01–0.10

**Table 4:** Chemical analysis of white sugar.

Parameter	Description	Methodology	Typical Range/Value [8,35]
Polarization	Optical rotation of sucrose	ICUMSA GS2/3-1 (2011) [61]	≥99.7 °Z
Moisture	Water content	ICUMSA GS2/1/3/9-15 (2007) [62]	≤0.06%
Reducing substance	Glucose and fructose levels	ICUMSA GS2/3/9-5 (2011) [63]	≤0.04%
Color	White sugar solution color	ICUMSA GS2/3-10 (2011) [64]	≤45 IU_420_
Reflectance	Whiteness and purity of sugar crystals	ICUMSA GS2-13 (2011) [65]	≥99.0%
Conductometric ash	Determines mineral content	ICUMSA GS2/3/9-17 (2011) [66]	≤0.027%
Sulfite content	Sulfite levels	ICUMSA GS2/1/7/9-33 (2011) [67]	≤10 mg/kg
Insoluble matter in water	Insoluble impurities	ICUMSA GS2/3/9-19 (2007) [68]	≤20 mg/kg
Turbidity	Cloudiness of white sugar solution	ICUMSA GS2/3-18 (2013) [69]	≤30 IU
pH	Acidity or alkalinity sugar water solution	ICUMSA GS1-23 (2009) [70]	6.5–8.0
Ferromagnetic contaminants	Ferromagnetic materials	PN-A-74855-10:1987 [71]	≤0.5 mg/kg

## Data Availability

Data presented in this study are available on request from the corresponding author.
